# Prevalence of Mental Health Complaints Among Performing Arts Students Is Associated With COVID-19 Preventive Measures

**DOI:** 10.3389/fpsyg.2021.676587

**Published:** 2021-06-15

**Authors:** Janine H. Stubbe, Annemiek Tiemens, Stephanie C. Keizer-Hulsebosch, Suze Steemers, Diana van Winden, Maurice Buiten, Angelo Richardson, Rogier M. van Rijn

**Affiliations:** ^1^Codarts Rotterdam, University of the Arts, Rotterdam, Netherlands; ^2^PErforming Artist and Athlete Research Lab (PEARL), Rotterdam, Netherlands; ^3^Department of General Practice, Erasmus MC University Medical Centre Rotterdam, Rotterdam, Netherlands; ^4^Rotterdam Arts and Sciences Lab (RASL), Rotterdam, Netherlands; ^5^Department of Human Movement Sciences, Vrije Universiteit Amsterdam, Amsterdam Movement Sciences, Amsterdam, Netherlands

**Keywords:** COVID-19, performing arts, dance, circus, musical theatre, mental problems

## Abstract

The aim of this study was to investigate the effect of COVID-19 preventive measures on the mental health of performing arts students. In a prospective cohort study, performing arts students (*N* = 213) from Codarts Rotterdam, University of the Arts, were invited to monitor their health during one academic year (September 2019–May 2020). Every month, students completed items on mental health complaints, stress, and sleep quality. Chi-square tests and repeated-measures ANOVA with deviation contrasts were performed to investigate whether COVID-19 preventive measures were associated with changes in mental health complaints, stress scores, and sleep quality. During the COVID-19 lockdown, subjective mental health, Mental Health Inventory-5 (MHI-5), and items on loneliness were additionally completed by the respondents. A total of 98 students (46.0%) were included in the analyses. The 3-month prevalence of mental health complaints was significantly higher during the COVID-19 lockdown compared to the two pre-COVID-19 periods (*p* < 0.001). Mean stress scores were significantly lower for February (35.20) and March (36.41) when compared to the overall mean (40.38). Sleep quality scores (mean) were significantly higher for April (6.90), and May (6.89) when compared to overall mean (6.58). Furthermore, at least 75.5% of the participants dealt with moderate to very severe loneliness in all 3 months during the COVID-19 lockdown. During lockdown, performing arts students perceived less stress and their sleep quality increased. However, the prevalence of mental health complaints increased. Besides, 3 out 4 students dealt with moderate to very severe loneliness.

## Introduction

The coronavirus disease 2019 (COVID-19) has resulted in a global pandemic with unprecedented consequences. Governments are forced to implement measures to reduce the rapid spread of the disease. These measures range from recommendations on an individual level (i.e., using a mask, washing hands frequently, avoiding public contact, maintaining a safe distance between people) to strict collective prevention measures such as confinement and social isolation (Adhikari et al., 2020; Arefi and Poursadeqiyana, 2020).

A growing number of studies have investigated the psychological impact of COVID-19 on the general population (i.e., Fitzgerald et al., 2020), and specific target groups; for example, athletes (i.e., Pillay et al., 2020), children (i.e., Ghosh et al., 2020), adolescents (i.e., Imran et al., 2020), medical staff (i.e., Luo et al., 2020), older people (i.e., Sepúlveda-Loyola et al., 2020), patient groups (i.e., López-Fando et al., 2020), performing arts professionals (Spiro et al., 2021), and students (Elmer et al., 2020). The results have shown that mental health problems (i.e., anxiety, depression, and stress) are common reactions to the COVID-19 pandemic (Rajkumar, 2020; Kunzler et al., 2021).

To our knowledge, no study to date has investigated the effect of COVID-19 on mental health complaints of performing arts students. However, a recent cross-sectional study investigated the impact of the COVID-19 lockdown on the health, well-being, and livelihoods of professional performing artists (Spiro et al., 2021). In this cross-sectional study, data from a survey was analysed both quantitatively and qualitatively. The quantitative analysis of this study showed that 63% of the respondents reported feeling more lonely, and 85% reported feeling more anxious as a result of the public health situation. In their own words professional performing artists described that the lockdown had negative implications for health and well-being, including anxiety, low mood, worsening, or new symptoms of depression, unstable or fluctuating moods, and poor sleep.

In students, COVID-19-specific worries, isolation in social networks, a lack of interaction and emotional support, and physical isolation have been related to mental health issues (Elmer et al., 2020). Compared to the general student population, performing arts students might even be more affected by social isolation. Whereas, switching to online teaching is a good alternative for the general student population, for performing arts students classroom teaching is essential to interact with teachers and students to work on their technical and aesthetical abilities, as well as working together on specific performances. Furthermore, performing arts students spend most of their time at school. School days from 9 am to 6 pm are the rule rather than the exception.

A cross-sectional study among elite and semi- elite athletes found that suspending seasons and cancelling competitions, as a result of the COVID−19 lockdown, cause significant grief, stress, anxiety, frustration, and sadness (Pillay et al., 2020). The psychological impact of COVID-19 on performing arts students might be—just as in athletes—increased by the disruption of one's normal routine and social support network.

In the Netherlands, both personal and collective preventive measures have been taken to slow down the disease spread and prevent health systems from becoming overwhelmed. On March 15, 2020, the government closed public places such as educational institutions, bars, restaurants, cafés, and coffee shops. The peak of the epidemic was from late-March to early-April 2020. Performing arts students in the Netherlands were banned from all performing arts activities (classes, rehearsal, and performances), and were unable to attend classes at school from March 16 to June 15, 2020 (13 weeks). This study aims to investigate the effects of COVID-19 preventive measures on the mental health of performing arts students using a prospective design with monthly follow-up. We hypothesise that mental health complaints, stress, and sleep quality have been negatively affected by the COVID-19 preventive measures.

## Materials and Methods

### Participants

All first-, second-, and third-year students (*N* = 213) of the Bachelors in Dance, Dance and Education, Circus Arts, and Music (specialisation Music Theatre) from Codarts Rotterdam (University of the Arts) were prospectively followed on a monthly basis during one academic year (September 2019–May 2020). This monitoring is part of the students educational program them to monitor their health and be more effective in learning and performing and taking responsibility for their own personal development. All students are enrolled in a 4-year educational program, resulting in a Bachelor of Arts degree. Fourth-year students are not included in this study because they do not complete questionnaires as a result of their internships. All students were informed about the purpose and procedure of this study and provided written informed consent. The study was approved by the Medical Ethics Committee (MEC-2019-0163) of the Erasmus University Medical Centre Rotterdam, the Netherlands. For the analysis of this study, we selected the students who completed at least all three questionnaires during the COVID-19 lockdown (March, April, and May 2020).

### Data Collection Procedure

The Performing artist and Athlete Health Monitor (PAHM) was used to monitor the mental health of performing arts students (Stubbe et al., 2018; Karreman et al., 2019; Van Winden et al., 2019). Every month, students were invited to complete a questionnaire concerning their physical and mental health during one of their theory classes. A reminder was sent to the students that were not present during these classes, and a second reminder was sent to all students who did not respond to the questionnaire after 1 week.

### Measuring Instruments

PAHM incorporates several questionnaires and items and is accessible from personal computers, tablets, and phones. Table 1 gives an overview of the items that we used in this specific study.

**Table 1:** Overview of the items included in the questionnaires before and during the COVID-19 lockdown.

	**Pre-COVID-19 period (Sep-Feb 2020)**	**COVID-19 period (Mar-May 2020)**
Mental health complaint (prevalence)	X	X
Stress (0–100)	X	X
Sleep quality (1–10)	X	X
Mental health (1–5)		X
MHI-5 (0–100)		X
Loneliness (0–11)		X
- Sum score		X
- Emotional loneliness		X
- Social loneliness		X

Several questionnaires and items were assessed before (September 2019–February 2020), and during the COVID-19 lockdown (March 2020–May 2020). Firstly, the Oslo Sports Trauma Research Centre (OSTRC) Questionnaire on Health Problems (Clarsen et al., 2014; Stubbe et al., 2018; Van Winden et al., 2019, 2020); was included to assess the most severe mental health complaint. The OSTRC questionnaire consists of four key questions on the consequences of health problems for participation, training volume, and performance, as well as the degree to which the student perceived symptoms. Each question of the OSTRC was scored with a four- or five-point scale, ranging from 0 (respectively: no problem, no reduction, no effect and no symptoms) to 25 (cannot participate at all or severe symptoms). The severity of the health problem was calculated by the sum score of the four questions (0: no health problem −100: cannot participate at all due to severe health problems), according to the method proposed by Clarsen et al. (2014). If the severity score was higher than zero, a health problem was registered and the student was asked whether the health complaint was an injury, mental health complaint, illness, or other problem. Second, a visual analogue scale (VAS) was used to assess stress with scores ranging from 0 (no stress) to 100 (extreme amount of stress). The VAS is frequently used in stress assessment and several validity studies have highlighted its psychometric properties. The VAS is at least as discriminating as other stress scales (i.e., 14 items Perceived Stress Scale) (Lesage et al., 2011), shows satisfactory inter judge reliability (Lesage et al., 2011, 2012) and reliability (Lesage et al., 2009).

Third, sleep quality was measured using a single-item (i.e., How would you rate your sleep quality overall in the past 4 weeks?) on an 1–10 nummeric rating scale (NRS), where 1 is worst possible sleep and 10 best possible sleep. The sleep quality NRS have demonstrated strong psychometric properties, including reliability, validity, and responsiveness and is correlated with relevant aspects of the frequently used Medical Outcome Study (MOS) sleep scale (Martin et al., 2009, Cappelleri et al., 2009).

During the COVID-19 lockdown, several additional items were included in the survey. First, subjective mental health was measured with one single question on a scale ranging from one (excellent) to five (poor) (i.e., In the past 4 weeks, would you say your mental health is...), which was based on the first question of the 36-item Short-Form Health Survey (SF-36) (Ware and Sherbourne, 1992). Second, the five-item Mental Health Inventory (MHI-5)—a subscale of the 36-item Short-Form Health Survey (SF-36)—was applied to assess mental health (Ware and Sherbourne, 1992). The MHI-5 comprises five items with six possible responses ranging from “all the time” (1 point) to “none of the time” (6 points). The final MHI-5 score is calculated by summing up the item scores and transforming this score to a scale varying from 0 to 100, with lower scores indicating more severe mental complaints. In the present study, a cut-off point of 60 was used (Thorsen et al., 2013). Participants with a MHI-5 score ≤ 60 were classified as mentally unhealthy and participants with a MHI-5 score > 60 were classified as mentally healthy. Third, loneliness was assessed by the eleven-item loneliness scale (De Jong Gierveld and Van Tilburg, 1999), with response categories of “yes,” “more or less,” and “no.” The item scores of the loneliness scale were dichotomized in agreement with the scaling procedure (yes = 1; more or less = 1; no = 0). The total loneliness scale score was computed as the sum of the dichotomized items, ranging from 0 (absence of loneliness) to 11 (extreme loneliness). The following four categories were used: no loneliness (score <3), moderate loneliness (score 3–8), severe loneliness (score 9 or 10), and very severe loneliness (score 11) (Elmer et al., 2017).

### Statistical Analysis

Statistical analyses were conducted using SPSS (SPSS, V24.0) and statistical significance level was set at an alpha level > 0.05. Descriptive statistics were used to describe characteristics of the study population (i.e., age, gender, educational program) using mean and standard deviation (SD) or number and percentages (%). The response rate was calculated for the total student group (number of completed questionnaires/total number of questionnaires send out to students ^*^ 100).

Based on the OSTRC questionnaire, the 3-month prevalence of mental health complaints was calculated by dividing the number of students who reported a mental health complaint during a 3-month period by the number of respondents in the same 3 months. Chi-square tests were performed to investigate whether the differences between the two pre-COVID-19 periods—September to November and December to February—and the COVID-19 lockdown (March to May) were statistically significant. Besides, means and SD per month were calculated for stress and sleep quality. Repeated-measures ANOVA with deviation contrasts were performed to investigate whether stress and sleep quality scores in any of the pre-COVID-19 months (September-February) and the COVID-19 lockdown (March-May) significantly deviated from the overall mean.

Means and SD or numbers and percentages were calculated monthly during the COVID-19 lockdown (March-May) for subjective mental health, MHI-5, and the loneliness scale.

## Results

### Participants and Response Rates

A total of 213 students were invited to participate in this study. Nine students did not sign the informed consent and were excluded from the analyses. A total of 106 students did not complete all three questionnaires during the COVID-19 lockdown and were excluded from the analyses. This resulted in 98 students included in the analyses (46.0%). Of these, 32 students were enrolled in the bachelor course in dance, 32 in dance teacher, 25 in circus, and 9 in music theatre. The mean age of the students was 19.89 (SD = 2.0). Overall, 75.5% of the students were female. The 98 enrolled students were monitored on a monthly basis during one academic year (9 months) resulting in a total of 882 questionnaires (98 students ^*^ 9 months) sent during the academic year. Of these, 859 were completed leading to a response rate of 97.4% across the academic year.

### Mental Health Complaints According to the OSTRC

The 3-month prevalence of mental health complaints was 21.4% (September-November) and 24.5% (December-February) during the two pre-COVID-19 periods and 27.6% (March-May) during the COVID-19 lockdown. This difference was statistically significant [*X*^2^_(1)_ = 11.73, *p* < 0.001 and *X*^2^_(1)_ = 19.45, *p* < 0.001].

### Stress

**Figure 1:**
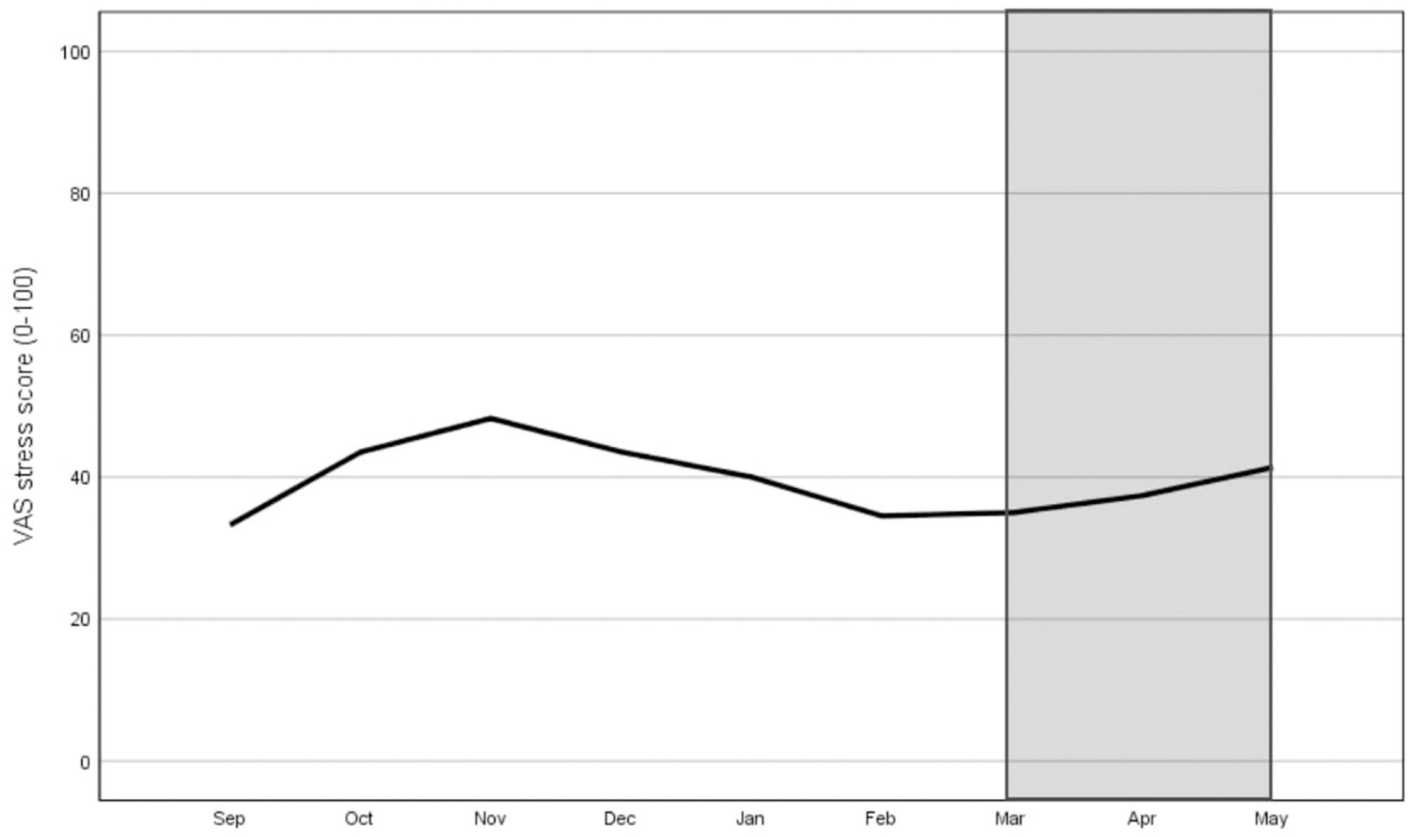
Monthly stress score (VAS scale ranging from 0 to 100) during the pre-COVID-19 period (Sep-Feb) and during the COVID-19 lockdown (Mar-May: grey area).

Figure 1 displays the scores on the VAS stress scale. The average stress score was 40.38 during the pre-COVID-19 period and 37.66 during the COVID-19 lockdown. The assumption of sphericity was violated by Mauchly's Test of Sphericity [$\chi_{\left(35\right)}^{2}$ = 84.45, *p* < 0.001]. As a result, the Greenhouse-Geisser correction was used. There was a significant effect of time on VAS stress scores: *F*_(6.45, 496.84)_ = 5.61, *p* < 0.001. Tests of within-subjects contrast showed that VAS stress scores were significantly higher for October (M = 43.32, SD = 23.36) and November (M = 48.20, SD = 24.05) and significantly lower for February (M = 35.20, SD = 23.09) and March (M = 36.41, SD = 23.39) when compared to the overall mean.

### Sleep Quality

**Figure 2:**
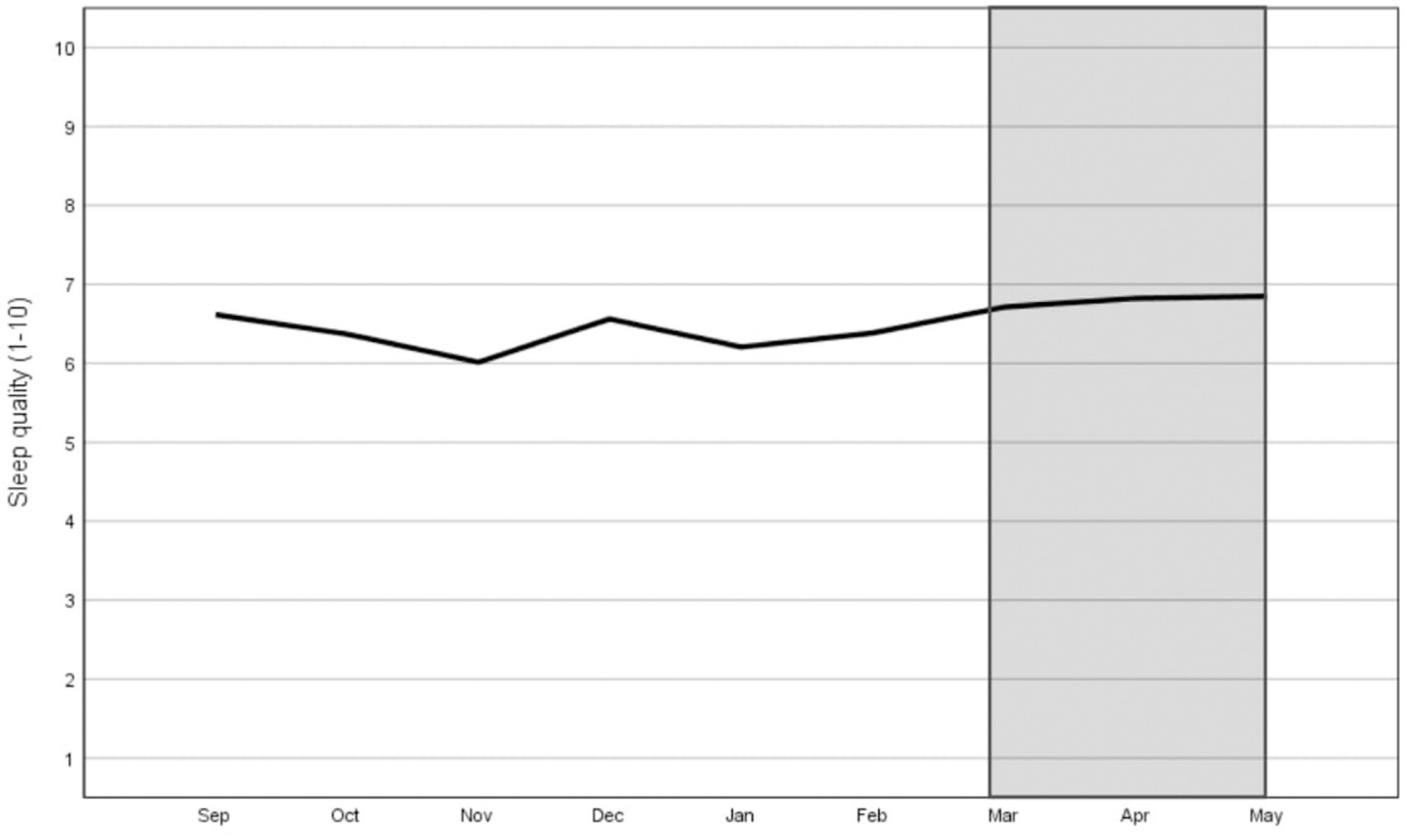
Monthly sleep quality score (ranging from 1 to 10) during the pre-COVID-19 period (Sep-Feb) and during the COVID-19 lockdown (Mar-May: grey area).

Figure 2 shows the sleep quality scores on a ten-point scale. The average sleep quality score was 6.58 throughout the academic year. The average score was highest in April and lowest in November. The average sleep quality score was 6.44 during the pre-COVID-19 period and 6.87 during the COVID-19 lockdown. The assumption of sphericity was violated by Mauchly's Test of Sphericity [$\chi_{\left(35\right)}^{2}$ = 102.482, *p* < 0.001]. Therefore, the Greenhouse-Geisser correction was used. There was a significant effect of time on sleep quality scores: *F*_(6.01, 432.92)_ = 3.83, *p* < 0.001. Tests of within-subjects contrast showed that sleep quality scores were significantly lower in November (M = 6.12, SD = 1.76) and January (M = 6.33, SD = 1.39) and significantly higher for April (M = 6.90, SD = 1.54) and May (M = 6.89, SD = 1.56) when compared to the overall mean.

### Additional Mental Health Items During COVID-19 Lockdown: Subjective Mental Health, Mental Health Inventory, and Loneliness

Subjective mental health rating scores were 3.07 ± 0.87 in March, 3.16 ± 0.98 in April, and 3.24 ± 0.89 in May. The results showed that 27.6% of the participants were mentally unhealthy in March, 32.7% in April, and 35.7% in May. At least 75.5% of the participants dealt with moderate to very severe loneliness in all 3 months.

## Discussion

In this study, we investigated the change in mental health, stress levels, and sleep quality of performing arts students during the COVID-19 pandemic. We compared the prevalence of mental health complaints, stress levels, and sleep quality during COVID-19 lockdown with the prevalence, stress and sleep scores in the 6 months prior to the lockdown. This within-person comparison showed that the prevalence of mental health complaints of performing arts students was negatively affected by the strict collective prevention measures taken by the Dutch government.

Our results are in agreement with results from a study including undergraduate students (Elmer et al., 2020). This Swiss study showed that undergraduate students became more depressed and slightly more anxious during the COVID-19 outbreak. Factors associated with this decline in mental health included living alone, as well as receiving less contact and support from one's personal network. Earlier empirical research has also highlighted the importance of social networks for health and well-being (Kawachi and Berkman, 2001; Elmer et al., 2017). The absence of social support might play an important role in explaining the increase in performing arts students' mental health complaints. The majority of these students come from abroad and are living on their own for the first time, away from family and friends. Due to the lockdown restriction, these students were isolated from their social support network, and some of them were forced to live in self-isolation for the majority of the day during a three-month period. This is challenging, and our study showed that at least three-quarters of participants dealt with moderate to very severe loneliness during this period. Furthermore, it is suggested that performing artists are more strongly affected than the population at large by the special conditioning of the COVID-19 pandemic, because they view performing as a basic necessity. Accordingly, being deprived of this is stressful to them (Primov-Fever et al., 2020).

Our study did not find a significant negative association between stress levels, sleep quality and the restrictions during the COVID-19 lockdown. This might be explained by the fact that students perceived both positive and negative changes due to the situation. This is in agreement with a previous study showing that some students reported that the crisis situation affected their lives positively regarding a reduced fear of missing out and sense of competition among students (Elmer et al., 2020).

### Strengths and Limitations of the Study

To our knowledge, this is the first study to investigate the effects of COVID-19 regulations on the mental health of performing arts students. Although the sample size of our study is relatively small, the response rate is rather high, and the presented data is accurately measured. Therefore, we believe that the results are representative for this specific target population. A major strength of our study is the use of a prospective design with monthly follow-ups. This design enabled us to assess the association between an exposure (i.e., COVID-19 regulations) and an outcome (i.e., mental health).

Besides these strengths, our study has several limitations. First, we used student-reported outcomes to assess mental health problems, which produced subjective data. We recommend the inclusion of a follow-up from the medical staff (e.g., clinical psychologist) during the data collection period in future studies to assess detailed diagnostic information regarding the registered mental health problems (i.e., diagnose, severity, comorbidity). Second, students were only able to register their most severe health problem, whereas research has shown that mental health problems often co-exist with physical problems (Adam et al., 2004; Mainwaring and Finney, 2017).

Therefore, it might be difficult for students to address them separately, which may have resulted in under-estimating the prevalence of all mental health problems. However, we used this arbitrary method both before and during the COVID-19 lockdown, and therefore our significant increase in mental health problems cannot be explained by this data collection method. Under-estimation of mental health problems can also be caused by the heightened stigma around mental health. The fear of being excluded due to mental health problems has been illustrated in athletic (Bauman, 2016) and dance populations (Karreman et al., 2019). Due to these reasons, the prevalence of mental health complaints in our sample cannot be compared with the prevalence found in other target populations. Finally, to assess the most severe mental health complaint, the OSTRC questionnaire on health problems was used (Clarsen et al., 2014). This questionnaire was validated in elite athletes but a comprehensive assessment of the psychometric properties of the OSTRC questionnaire within performing artists is lacking. Kenny et al. (2018) found a moderate agreement between third-party and self-reported injury registration, using the OSTRC questionnaire, in a population of pre-professional dancers (Kenny et al., 2018). Despite the fact that this questionnaire is regularly used in a population of performing artists (Kenny et al., 2018; Stubbe et al., 2018; Van Winden et al., 2019, 2020) future investigations assessing the psychometric properties of the OSTRC questionnaire in such a population are needed.

## Conclusion

The prevalence of mental health problems in performing arts students significantly increased during the COVID-19 lockdown, during which a total of 27.6% students sustained a health problem. At least 75% of the participants dealt with moderate to very severe loneliness in all 3 months during the COVID-19 lockdown.

At the time of writing, the COVID-19 crisis is still unfolding, and more research is needed to grasp the effects of COVID-19 on health issues in performing artists in the long term. We hope that our results encourage replications of the study in different countries, subgroups of performing arts (music, dance, and circus), and skill levels (amateur, pre-professional, and profession level), which will increase collective knowledge about successful interventions to enhance performing artists' mental health during the COVID-19 pandemic and beyond.

## Data Availability Statement

The raw data supporting the conclusions of this article will be made available by the authors, without undue reservation.

## Ethics Statement

The studies involving human participants were reviewed and approved by Medical Ethics Committee of the Erasmus University Medical Centre Rotterdam, the Netherlands. The patients/participants provided their written informed consent to participate in this study.

## Author Contributions

JS initiated the study and wrote the first draft of the manuscript. AT, MB, and AR performed the data collection. JS, AT, and RR analysed the data. All authors contributed to manuscript revision, read, and approved the submitted version.

## Conflict of Interest

The authors declare that the research was conducted in the absence of any commercial or financial relationships that could be construed as a potential conflict of interest.
